# Drug–Drug Interactions in Vestibular Diseases, Clinical Problems, and Medico-Legal Implications

**DOI:** 10.3390/ijerph182412936

**Published:** 2021-12-08

**Authors:** Giulio Di Mizio, Gianmarco Marcianò, Caterina Palleria, Lucia Muraca, Vincenzo Rania, Roberta Roberti, Giuseppe Spaziano, Amalia Piscopo, Valeria Ciconte, Nunzio Di Nunno, Massimiliano Esposito, Pasquale Viola, Davide Pisani, Giovambattista De Sarro, Milena Raffi, Alessandro Piras, Giuseppe Chiarella, Luca Gallelli

**Affiliations:** 1 Department of Law, Magna Graecia University of Catanzaro, 88100 Catanzaro, Italy; giulio.dimizio@unicz.it (G.D.M.); amalia.piscopo@gmail.com (A.P.); cicontevaleria@gmail.com (V.C.); 2 Department of Health Science, School of Medicine, University of Catanzaro, Clinical Pharmacology and Pharmacovigilance Unit, Mater Domini Hospital, 88100 Catanzaro, Italy; gianmarco.marciano@libero.it (G.M.); palleria@unicz.it (C.P.); lalumuraca@gmail.com (L.M.); raniavincenzo1@gmail.com (V.R.); roberta.roberti9@outlook.com (R.R.); desarro@unicz.it (G.D.S.); gallelli@unicz.it (L.G.); 3 Department of Primary Care, ASP 7, 88100 Catanzaro, Italy; 4 Department of Experimental Medicine L. Donatelli, Section of Pharmacology, School of Medicine, University of Campania Luigi Vanvitelli, 80123 Naples, Italy; giuseppe.spaziano@vanvitelli.it; 5 Department of History, Society and Studies on Humanity, University of Salento, 83100 Lecce, Italy; nunzio.dinunno@unisalento.it; 6 Department of Medical, Surgical Sciences and Advanced Technologies “G. F. Ingrassia”, University of Catania, 95121 Catania, Italy; massimiliano.esposito9@gmail.com; 7 Unit of Audiology, Department of Experimental and Clinical Medicine, Regional Centre of Cochlear Implants and ENT Diseases, Magna Graecia University, 88100 Catanzaro, Italy; pasqualeviola@unicz.it (P.V.); davidepisani@gmail.com (D.P.); chiarella@unicz.it (G.C.); 8 Research Center FAS@UMG, Department of Health Science, University of Catanzaro, 88100 Catanzaro, Italy; 9 Department of Biomedical and Neuromotor Sciences, University of Bologna, 40126 Bologna, Italy; alessandro.piras3@unibo.it; 10 Medifarmagen SRL, University of Catanzaro, 88100 Catanzaro, Italy

**Keywords:** polytherapy, elderly, vestibular disease, vertigo, drug interactions, clinical practice, clinical risk management, safety of care, medico legal aspects

## Abstract

Peripheral vestibular disease can be treated with several approaches (e.g., maneuvers, surgery, or medical approach). Comorbidity is common in elderly patients, so polytherapy is used, but it can generate the development of drug–drug interactions (DDIs) that play a role in both adverse drug reactions and reduced adherence. For this reason, they need a complex kind of approach, considering all their individual characteristics. Physicians must be able to prescribe and deprescribe drugs based on a solid knowledge of pharmacokinetics, pharmacodynamics, and clinical indications. Moreover, full information is required to reach a real therapeutic alliance, to improve the safety of care and reduce possible malpractice claims related to drug–drug interactions. In this review, using PubMed, Embase, and Cochrane library, we searched articles published until 30 August 2021, and described both pharmacokinetic and pharmacodynamic DDIs in patients with vestibular disorders, focusing the interest on their clinical implications and on risk management strategies.

## 1. Introduction

The function of balance is regulated by the vestibular system. The main subdivision of this system, includes a peripheral portion (the posterior labyrinth, in the inner ear, and the vestibular portion of the eighth cranial nerve) and a central portion (vestibular nuclei, the oculomotor nuclei, the vestibulo–ocular reflex tracts, the cerebellum, the brainstem reticular formation, the area postrema, and other associated areas, including many refined connections with cognitive areas) [1]. Both, peripheral and central, acute and chronic impairment of the vestibular system determine the appearance of vertigo/dizziness with other symptoms (e.g., postural imbalance, nausea, vomiting, and in some particular clinical pictures, also sensorineural hearing loss and tinnitus) [2,3,4].

Vertigo is a highly disabling disorder that impairs the daily activities, especially in elderly patients, exposing this population to the risk of falling.

In elderly, a very significant complication is represented by both multiorgan impairment and alteration of pharmacokinetic parameters. Therefore, several factors—(i) polytherapy, (ii) changes in body composition, (iii) renal or liver impairment—play a role in the development of adverse drug reactions (ADRs) [5] and also induce an increased vulnerability of older patients [6,7].

In addition, polytherapy can increase the risk of drug–drug interactions (DDIs) and drug–disease interactions that are involved in falls, longer time of hospitalization, and even death [6,8].

Many pharmacological treatments modulate the activity of neurotransmitters, neuromodulators, and voltage-gated channels involved in the modulation of neuronal excitability.

Among all of these drugs, cinnarizine is the first-line therapy for the management of some kind of vertigo. It may act on the peripheral vestibular system with several mechanism [9,10,11,12] able to induce both an anti-vasoconstrictor activity and a decrease in blood viscosity of the inner ear’s circulatory system [11]. Usually in clinical studies, cinnarizine is used at different doses, from 15 mg tid to 150 mg od; however, the common dosage is 25 mg tidor 75 mg od, up to a maximum of 225 mg [13]. Today, cinnarizine is administered with dimenhydrinate, an histamine (H1) receptor antagonist and phosphodiesterase inhibitor in the vestibular nuclei and the vomiting center, with anti-vertigo and anti-emetic effects [14,15].

Different mechanisms of action, related to the drug use, reduce the strength of symptoms (e.g., vestibular suppressants) or modify the underlying disease processes that have led to the development of symptoms (e.g., calcium channel antagonists in the case of vestibular migraine).

Previously, we reported that DDIs represent a risk factor in poly-treated patients, because they can induce the development of ADRs [16,17,18,19]. In the present review we assessed the possibility of DDIs and ADRs in elderly people with vestibular disease.

## 2. Methods

The PubMed, Embase and Cochrane library databases were searched for articles published until 30 August 2021 in English language, in agreement with our recent papers [20,21].

Secondary search included articles cited in reference lists identified by the primary search. Records were first screened by title/abstract before full-text articles were retrieved for eligibility evaluation. Remaining articles were then subject to a citation search of all reference lists. Papers were deemed eligible if they included any form of words: “adverse drug reaction”, “drug interactions”, “polytherapy”, “vestibular disease/s”, “systemic diseases”. All citations were downloaded into Mendeley, and duplicates deleted. GM and PV screened all articles by title/abstract to determine their eligibility and RR reviewed a random sample of 20% to evaluate the reliability of the selection process. To avoid a bias of exclusion, the full text articles were retrieved following first round exclusions and were also subject to two independent eligibility reviews (CP 100%, LM 20%), this time with perfect agreement. The studies evaluated as eligible were enclosed in the present review. We excluded manuscript without full text and without indications of adverse drug reactions. Moreover, we also excluded manuscript not in English language. MR revised the manuscript elucidating the physiological mechanisms.

### 2.1. Drugs Used in the Management of Peripheral Vestibular Vertigo

#### 2.1.1. Betahistine

Betahistine dihydrochloride is an oral drug, a strong antagonist of histamine H3 receptors and a weak agonist of H1 receptors [22]. Betahistine increases vasodilation and restores microcirculation [23] and blood flow, but also facilitates vestibular compensation and inhibits spike generation in lateral and medial vestibular nuclei [24]. Its main clinical indication is represented by MD (Meniere’s disease) [22], but it may be also used in BPPV (benign paroxysmal vertigo, in selected patients) [25], vestibular neuritis, and other cases of peripheral vestibular vertigo [24].

A systematic review by Murdin et al. [26] assessed betahistine’s efficacy in the treatment of vertigo despite low level of evidence. According to the BEMED trial performed by Adrion et al., low and high dose betahistine seem to be not superior to placebo on reducing the incidence of attacks caused by MD. Both placebo and betahistine lowered attack ratio and this result was probably related to natural history of MD [27]. Another systematic review showed no efficacy of betahistine in MD, sustaining the need of high quality studies [28].

Monoamine oxidases (MAOs) A/B are responsible of betahistine metabolism, therefore MAO inhibitors may increase its concentration [23,29], inducing the development of few ADRs [23,27]: (Table 1, Table 2, Table 3, Table 4, Table 5, Table 6, Table 7 and Table 8).

#### 2.1.2. Diuretic Therapy

Diuretic drugs are commonly used for the treatment of hypertension and heart failure. Their action is mainly characterized by fluid excretion [217].

Endolymphatic hydrops plays a key role in the pathogenesis of MD. Therefore, fluids reduction is a rational strategy of treatment. Diuretics showed a certain efficacy in the control of hearing loss and vertigo, especially in patients with non-advanced disease. However, further analysis demonstrated that they didn’t prevent a long-term deterioration of hearing [41]. A systematic review by Crowson et al. [218] stated that diuretics are more effective in the prevention of vertigo in patient with MD, with a lower action on hearing outcomes.

Hydrochlorothiazide/triamterene, acetazolamide, spironolactone are the most important diuretics used in MD, whereas loop diuretics may be employed, but need a close monitoring of ADRs [22,41,219].

Hydrochlorothiazide and acetazolamide are carbonic anhydrase inhibitors, whereas triamterene and spironolactone have a potassium sparing mechanism (Table 1).

#### 2.1.3. Acetazolamide

According to magnetic resonance imaging (MRI) study, Sepahdari et al. documented that acetazolamide reduces endolymphatic hydrops in subjects with MD. However, the action of acetazolamide could not be effective on hydrops in all patients and fluid could increase after therapy discontinuation [220].

#### 2.1.4. Hydrochlorothiazide

Hydrochlorothiazide is a thiazide-type diuretic. It exerts its action inhibiting sodium chloride co-transporter in the distal convoluted tubule [31,32]. Its role in MD is well described [219,221], and it can be commonly administered with triamterene [22,41] (Table 1, Table 2, Table 3 and Table 4).

#### 2.1.5. Triamterene

Triamterene, a potassium-sparing diuretic, inhibits the epithelium sodium channel (ENaC), avoiding sodium-potassium exchange [35,38]. Triamterene is often administered with hydrochlorothiazide since it enhances the effect of hydrochlorothiazide in lowering blood pressure and prevents hypokalemia. In MD the two drugs are often prescribed in a coformulation [22,35,41] (Table 1, Table 2, Table 3 and Table 4).

#### 2.1.6. Spironolactone

Spironolactone is a potassium-sparing diuretic. It is a mineralocorticoid receptor antagonist which blocks the consequential action of aldosterone on ENaC (Epithelium Sodium Channel). Spironolactone determines the excretion of chlorine, sodium and water, whereas potassium is retained [33,34,35], ad is recommended in MD [22,41].

In an experimental study, Degerman et al. showed that spironolactone blocks the phosphodiesterase 4 (PDE4) pathway, involved in the generation of endolymphatic hydrops [222].

Other studies described a positive effect of aldosterone on endolymphatic hydrops that is related to salt-reduced diet (generating an increase of aldosterone levels), that is part of the management of MD [223] (Table 1, Table 2, Table 3 and Table 4).

### 2.2. Vestibular Suppressants

#### 2.2.1. Benzodiazepines

Benzodiazepines (BDZ) are recommended in MD, VN and may be used for particular classes of patients in BPPV [22,25,41,42,56]. BDZ act on GABA_A_ receptor as allosteric modulators, increasing receptor affinity for its substrate, with an increase in GABA inhibitory activity [39,106]. Despite the effectiveness of BDZ being demonstrated [22,224,225,226,227], it has been suggested that they might impair compensation for vestibular lesions [228]. In fact, GABA_B_ activation seems to be involved in vestibular compensation after damage [229,230]. Diazepam, lorazepam, and clonazepam are more frequently used in the treatment of vestibular peripheral vertigo [22,228], due to short onset of action [41].

Lorazepam is metabolized by conjugation with glucuronic acid, diazepam is metabolized by CYP2C19 and CYP3A4 (part of its metabolites is conjugated) and clonazepam by CYP3A4 [43,45,46,81,82,84]. Inhibition/induction of these CYPs may generate increase/reduction of drugs concentrations (see Table 6).

BDZ main adverse events are neurologic symptoms (somnolence, motor difficulties, amnesia, difficulty concentrating, headache, vertigo, behavioral changes) and gastrointestinal symptoms. Incontinence, articular pain, hypersensitivity, hepatic damage, and hematologic alterations are rare. Abstinence, abuse, and tolerance are very common after BDZ administration. To avoid this kind of reaction physicians should use a gradual taper to discontinue BDZ, use a lower dosage. High dose and prolonged treatment are more frequently associated to these side effects [40,43,45,46,81,82,84].

#### 2.2.2. Anticholinergics

Anticholinergics are used as vestibular suppressants in MD, BPPV, VN, and BV [22,25,41,42,56]. The most common anticholinergics are scopolamine, atropine and glycopyrrolate [22,41]. These drugs, acting on acetylcholine (ACh), inhibit the ACh muscarinic receptor (mAChR) [22,40]. Clinical trials showed a certain efficacy of scopolamine (transdermal route) [55] and glycopyrrolate (oral route) [50] in the control of vertigo. ACh pathways seem to have a crucial role in the activity of the mammalian vestibular system. In an experimental model, Schneider et al. described that atropine and scopolamine inhibited vestibular spike responses in mice [231]. Other authors sustain that muscarinic and nicotinic receptors are involved in both inhibitory and excitatory responses [232] (Table 1, Table 2, Table 3 and Table 4).

Glycopyrrolate does not pass the blood–brain barrier; therefore, it has less neurologic side effects. It also shows minor ocular and cardiovascular events [40,49].

#### 2.2.3. Antihistamines

Antihistamines are commonly used as vestibular suppressants in the treatment of peripheral vestibular vertigo. Dimenhydrinate, meclizine and diphenhydramine (first-generation antihistamines) are the most common compounds. Diphenhydramine is a dimenhydrinate metabolite [22,25,40,41,56]. They exert their action blocking histamine receptor H_1_ [40].Cinnarizine, a calcium channel blocker, may be administered in combination with dimenhydrinate: The combination was more effective than monotherapy [14,233]. Phenothiazines are antipsychotic drugs that also act on histaminergic pathways and may be employed in the treatment of vertigo (e.g., promethazine) [41].An experimental model by Takatani et al. described an inhibitory action of diphenhydramine more selectively in medial vestibular nucleus (MVN) rather than lateral vestibular nucleus (LVN). Evidence about an important role of histaminergic pathways in vestibular system come from experimental models, showing a role of H1, H2, and H3 receptors. Counterintuitively, a high expression of H1 and H2 receptors is associated with a better vestibular compensation [62,234,235]. Not only the activity of histaminergic receptors seems to be effective in improving balance, but they also seem to modulate GABA pathways [234], reducing the concentration of the inhibitory neurotransmitter [235]. However, further studies are needed.

This positive role of histamine receptors seems to assess the need of a histaminergic agonist to treat vertigo, and betahistine (strong H3 antagonist and weak H1 agonist) was the main candidate [234]. However, trials described a superiority of betahistine compared with promethazine [236], but a minor effect than cinnarizine/dimenhydrinate [237]. Furthermore, a systematic review showed that evidence for betahistine in the treatment of peripheral vestibular vertigo has a low level of quality [26].Moreover, the clinical effectiveness of the other H1 antagonists was observed in other clinical trials [22,238,239,240].

Promethazine, meclizine, dimenhydrinate, and diphenhydramine are contraindicated in patients affected by lower respiratory tract infections, asthma [57,61,67,69].Physicians must pay attention also in cardiovascular diseases (promethazine) [69]. Dimenhydrinate should be used with caution in porphyria [57].Diphenhydramine is also contraindicated in pheochromocytoma, arrythmias (or risk of rhythm alterations) [61].

Diphenhydramine is a substrate and an inhibitor of CYP2D6 [92]. It is also metabolized with minor affinity by CYP1A2, CYP2C9 and CYP2C19 [92]. Meclizine reduces the expression of CYP2B10, 3A11, 1A2 [65], and it is metabolized by CYP2D6 [94].

Cinnarizine seems to be mainly metabolized by CYP2D family (CYP2D6) and CYP2B6, although other cytochromes are secondarily involved [88,89].

Meclizine is an agonist for murine constitutive androstane receptor (mCAR) and an inverse agonist for human constitutive androstane receptor (hCAR), involved in the regulation of hepatic enzymes [65].

#### 2.2.4. Other Antiemetics

Other drugs may be used for the management of symptoms related to vertigo. Among them, 5HT3 antagonists play an important role. This category includes ondansetron (the most used), granisetron, tropisetron, dolasetron, and palonosetron (second generation antagonist) [40]. In an experimental model by Zhang et al., serotonin had an important role in vestibular compensation: the increase of its levels was documented after semi-circular canal occlusion [241]. Ondansetron showed a better effect in the control of nausea and vomiting, but a minor efficacy in the treatment of peripheral vertigo in comparison with promethazine [238]. Nevertheless, an experimental model by Dyhrfjeld-Johnsen etal. showed a positive post-insult effect of ondansetron in vestibular neuritis [242]. Ondansetron may be also used in the management of MD [41]. It is metabolized by CYP1A2, CYP2D6 and CYP3A4 [40,75,99], and It is also a P glycoprotein (P-gp) substrate [100].

Metoclopramide is a pro-kinetic drug used in the management of nausea and vomiting. It acts on 5HT4 (agonist), 5HT3 (antagonist) and dopamine D_2_ (antagonist) receptors [40,71,72]. Dopamine may have an important role in vestibular activity. Jansen et al. showed a reduction of D_2_/D_3_ receptor in patients with bilateral vestibular failure. They speculate that this may obstacle vestibular compensation. Dopamine pathways are complex and regulate vestibular activity involving also cortex and basal ganglia [243]. An experimental model by Toro et al. showed a dopamine modulation of sensory hair cells activity acting in a paracrine fashion [244]. Metoclopramide had a similar efficacy to dimenhydrinate in reducing vertigo, nausea and vomiting in an emergency setting [245]. Metoclopramide (non-specific compound) is commonly prescribed in the management of MD, BPPV, VN, and other forms of vertigo [73].Metoclopramide is metabolized to mono-de-ethyl-metoclopramide at least in part by CYP2D6 isoform, and possibly CYP1A2 and CYP3A [97].

#### 2.2.5. Corticosteroids

Glucocorticoids have anti-inflammatory, anti-pyretic, and anti-allergic activity [133]. They modulate immune response through different mechanisms, for example, encoding pro-inflammatory cytokines IL-4, IL-10, IL-13, and TGFβ or inhibiting activation of nuclear factor (NF)-κB genes [246]. They act on the hypothalamic-pituitary-adrenal (HPA) axis [105,247]. Oral corticosteroids may be used in MD to reduce inflammatory response or to treat exacerbations. Evidence about their application is limited and their usage is based on expert opinion [248]. Even if dexamethasone, methylprednisolone and prednisone are the most common corticosteroids used [41], their role in VN is controversial. In fact, some authors suggest to start the treatment until 72h [56], whereas a systematic review failed to report a strong evidence [249]. Recent works described a role of corticosteroids in accelerating the recovery, without affecting prognosis [250]. Dexamethasone, methylprednisolone, and prednisone are metabolized by CYP3A4 [131,132,133,134]. Dexamethasone induces CYP3A4 and is both a substrate and inducer of P-gp [133,147]

#### 2.2.6. Other Treatment Options

Calcium antagonists other than cinnarizine (flunarizine and verapamil) may be useful in MD, especially in patients with migraine as prophylactic medications [219]. Flunarizine showed good results (clinical improvement) in a small court of 32 patients [251].

However, flunarizine efficacy was inferior to betahistine in MD in clinical trial [252].

A prostaglandin F2α analogue, latanoprost, may have a certain efficacy, since the F2 receptor has been found in inner ear. Intratympanic administration was associated with MD symptoms improvement in a small court, but further studies are needed [219].

Compounds mimicking and inducing glutathione peroxidase may exert anti-oxidant and anti-inflammatory effect in MD. Ebselen is a drug with these characteristics: Its administration in a phase 2 trial led to improvement of hearing and tinnitus in MD [219].

These drugs are not mentioned in MD guidelines [22].

Intratympanic gentamicin and intratympanic corticosteroids are two therapeutic options used in surgical setting [219,253].

## 3. Discussion

DDIs are an important concern in clinical practice. Assessing the relevance of DDIs is not easy since this task needs pharmaceutical, chemical, and clinical skills. Risk: benefit ratio should be always considered in the evaluation of DDIs. Minor interactions may be accepted, if necessary, when patients obtain a tangible improvement of their conditions [254]. Scott et al. defined deprescribing as “the systematic process of identifying and discontinuing drugs in instances in which existing or potential harms outweigh existing or potential benefits within the context of an individual patient’s care goals, current level of functioning, life expectancy, values, and preferences” [254]. This kind of approach is usually needed with elderly people, since they consume multiple medications, creating space for interactions [255]. Potentially inappropriate medications (PIM) generate an important part of DDIs. Their role is crucial because the reduction of their prescription or discontinuation may consistently reduce DDIs. When evaluating a patient, physician must be aware of all the drugs taken, consider the risk related to a specific drug and, above all, the real presence of a clinical indication. In fact, PIM will not only lead to no benefit, but will probably determine adverse events [254,255]. Therefore, physician should prescribe therapies according to guidelines and specific characteristics of patient.

Ageing is associated with multiple pathophysiological changes. An increase of adipose tissue may be observed in elderly people, especially women: then, the volume of distribution (Vd) of lipophilic drugs may be increased. Conversely, the reduction of body water is associated to a reduction of Vd for hydrophilic drugs. Minor differences in CYP450 metabolism were observed, whereas significant changes may be described in hepatic first pass metabolism (with a possible increase of bioavailability), α1acid glycoprotein (often increased), renal elimination (worsening). More data about transporters activity are needed [5,127]. These factors should be held in account in the management of elderly patients.

Sex differences should be evaluated. Vd is generally higher in men due to their larger body. However, women have a major percentage of fat compared to men, but the difference decreases with older age. This factor may be important in the evaluation of lipophilic drug distribution: For example, diazepam has a larger Vd in women; therefore, it may show an increased duration of its effect. Renal processes are faster in men, whereas CYP450 activity may vary greatly based on the single isoform, or the specific substance metabolized. CYP1A, CYP2D6 metabolism and hepatic conjugation are generally faster in men, whereas CYP3A activity seems to be quicker in women. These observations were often made on young and healthy subjects [256]. Specific and generalizable data about pharmacokinetics sex differences in elderly people are lacking. However, elderly females are predicted to have the lowest elimination rates, since they live longer than men and according to age-related increase of comorbidities, clearance reduction, and decrease of body size [256]. Another important statement is that there are important differences in adaptive homeostasis between males and females. Nevertheless, these sex-related distinctions seem to be lost with age [257]. Furthermore, sex-aging related differences in genomic instability were found, including a major tendency of DNA mutations, telomere attrition and sometimes methylation in men [258]. This assumption may affect gene expression related drug development, one of the present and future direction of pharmaceutic industry [259,260]. In addition, physicians should remember that females live longer than males, but have a higher risk of frailty and morbidity. Despite being less affected by comorbidities, males are characterized by a higher level of physiological dysregulation. Authors called it “male–female dysregulation frailty paradox” [257,261]. This concept may influence a therapy including multiple drugs.

Interaction in elderly may be various and unpredictable because this category of patients consumes multiple medications.

Drugs with anticholinergic (anticholinergic and antihistamines) effect should be avoided in patients suffering for urinary retention [262]. The concomitant consumption of cholinergic drugs is another contraindication to the use of this kind of therapy [54].In contrast, a difficulty in clinician’s decisions can be described in dementia or other neurologic pathologies related to urinary incontinence [263]. In fact, anticholinergic drugs (or compounds with anticholinergic effect) may worsen cognitive impairment. Crossing the blood-brain barrier (BBB) anticholinergics block muscarinic receptors M1 and M4. Furthermore, these drugs may interact with cholinesterase inhibitors, used in Alzheimer’s disease [198,263].

Diabetes is a difficult setting. If patients develop peripheral vestibular vertigo, metformin-glipizide and liraglutide are associated with dizziness. Other compounds may create difficulties in balance, affecting vision [264]. Diabetic neuropathy may cause urinary incontinence [265], since using anticholinergics to treat vertigo may be a right choice.

Acarbose is associated with flatulence and diarrhea, some of the most frequent adverse events of metoclopramide and other vestibular suppressants [72,138].

Cardiovascular setting is difficult since several interactions may happen (Table 5, Table 6 and Table 7). Despite some action of CYP450 enzymes on ACE inhibitors been described [102], they seem not to be part of important interactions, whereas angiotensin receptor blockers (ARB) may be involved in this kind of mechanism [101]. Nevertheless, the role of CYP450 enzymes on ARB metabolism is not always clear [103] and then predicting interaction is difficult.

NSAIDs are also associated with dizziness and then they may obstacle the treatment of vertigo [18].

Obstructive sleep apnea syndrome (OSAS) seems to be related to vertigo and to vestibular system. A reciprocal influence and neuroanatomical route between vestibular nuclei and orexinergic neurons have been reported. Sleep also stimulates neuroplasticity that helps vestibular compensation. Therefore, OSAS and sleep deprivation impair vestibular control, but also vestibular pathologies induce sleep disturbances [266,267]. Vestibular-evoked myogenic potentials may be used to evaluate vestibular lesions at the early stage of OSAS [268]. Furthermore, continuous positive airway pressure therapy in refractory MD patients with concomitant OSAS demonstrated an improvement of dizziness and audiometric testing [269]. According to these observations, the treatment of dizziness may improve OSAS. In this clinical situation, it is important to avoid drugs inhibiting respiratory system (e.g., opioids, barbiturates) [270]. Although characterized by a lower potential of respiratory depression, benzodiazepines may be associated with acute respiratory failure in patients with sleep apnea [40,271]. Physicians must remember it in the management of peripheral vestibular vertigo with concomitant pneumological pathologies. Furthermore, anticholinergics and antihistamines seem to reduce respiratory secretions generating rare and mild respiratory symptoms [40,49,53,54,61,214]. Atropine may generate respiratory depression [47].

Gastrointestinal symptoms or illness should be evaluated carefully. The induction of diarrhea by metoclopramide [74] may be troublesome in the management of electrolytic disturbances, gastrointestinal infections, colon cancer, and diverticulosis.

Oncologic patients may experience ototoxicity when treated with platinum compounds, surgery, or radiotherapy. This may also worsen or ingenerate vertigo [272]. Physician must pay attention to vertigo in oncologic patients since it may be the sign of brain metastasis. A series of red flags may add value to the clinical hypothesis: altered mental status, ataxia, headache, visual symptoms, seizure, and focal signs and symptoms [273]. Some of the drugs used for the management of peripheral vestibular vertigo may generate headache (e.g., ondansetron, triamterene, benzodiazepines, betahistine) [23,36,45,75,76,80,82] and blurred vision (e.g., antihistamines, anticholinergics) [274], therefore clinician must distinguish these side effects from cancer spread.

The susceptibility to infection in the elderly is increased by immune senescence as well as altered skin and mucosal barrier function [275].Among antibiotics, macrolides, aminoglycosides, and glycopeptides may generate ototoxicity, worsening clinical status of patients with peripheral vestibular vertigo [276].

An association between osteoporosis and BPPV has been reported, mainly in women [277]. Use of corticosteroids in this clinical setting may increase the risk of bone fracture [278]. Bisphosphonates undergo negligible liver metabolism [40], so no important interactions are expected. In the past, episodic vertigo after bisphosphonates intravenous administration was described [279]. On the other side, vertigo may increase the risk of falls in elderly people with osteoporosis, raising the possibility of fracture [280].

Rheumatology may be another field of interactions. For example, triamterene, anticholinergics, and antihistamines are associated with dry mouth [36,40,53,57,61], that could be an important issue in patients with Sjogren’s syndrome (SS) [281]. Hydrochlorothiazide may cause skin manifestation (common in rheumatologic pathologies) and SLE exacerbations [31,282,283]. Hydrochlorothiazide and triamterene may generate hyperuricemia, that would be a trouble in the management of gout [36,80,284].

Liver and kidney function is often altered in elderly people. CYP450 function is reduced in elderly, liver volume and blood flow decrease with age, similarly to immune response. An increased susceptibly to autoimmunity and hepatic infections is another major concern. All these factors may reduce tolerance to treatment [285]. A declination of glomerular filtration rate and a decreased number of functional glomeruli (alongside other anatomical alterations) are typical of aging and senescence [286]. Therefore, physicians must pay attention to vertigo treatment effect on liver and kidney status (Table 2, Table 3 and Table 4), especially if administered with other nephrotoxic or hepatotoxic medications [276,287].

Furthermore, hepatic cirrhosis may degenerate in hepatic encephalopathy [288] and then the use of CNS depressants drugs (see the sections before) should be avoided.

Another important chapter is related to nutraceuticals for the treatment of peripheral vestibular vertigo (e.g., *Ginkgo biloba,* lemon balm, ginger, citicoline, sage). These substances are not properly recommended in guidelines [22,25,56], but are often used in clinical practice. Nutraceuticals are usually safe compounds, but adverse events and interactions (including CYP450 enzymes) were reported [289,290,291,292,293,294,295,296].

Pharmacogenomics could be used to evaluate the individual response for each treatment. Therefore, clinicians should be aware of this kind of genetic variability to perform specific analysis (genotype) and adequate dosage to the result. However, non-genetic factors may generate a phenomenon called phenoconversion [297], creating a mismatch between genetic polymorphisms and the real capacity to metabolize drugs (Table 9).

## 4. Medico Legal Aspects: A Multidisciplinary Approach

DDI is a rising phenomenon due to the ageing of the population and the consequent increasing number of patients with comorbidities requiring multiple drug use [323]. For example, multiple drug use (polypharmacy) is common in diabetes mellitus (DM) patients [324]. DDIs are potentially harmful to patients, so full information is required also to improve the safety of care and to avoid malpractice claims. An essential requirement before administering or prescribing any substance is to adequately inform the patient of the expected benefits and any risks of the proposed activity. The Italian Law No. 219—“Provisions for informed consent and advance treatment directives”—specifically defines the issue of information and consent. In observance of the principles referred to in Articles 2, 12, and 32 of the Constitution and Articles 1, 2, and 3 of the Charter of Fundamental Rights of the European Union, the Law No. 2019 of 22 December 2017 not only stipulates that “no medical treatment shall be initiated or continued unless the person concerned has given free and informed consent, except in cases expressly provided for by law”, but also states that “The communication time between the doctor and patient constitutes care time”. Although legislation differs between countries, the promotion of a close care relationship based on informed consent should be widely considered as a goal of treatment, which requires a communicative method appropriate to the patient’s condition and abilities, which can be critical in the elderly population [325]. Informed consent to pharmacological treatment should not be limited to provide a correct information about the risk:benefit ratio of the drug but should be extended on the side effects and their frequency. Clinicians should sensitize patients to alert them if any abnormal symptom appears, in order to modify current polytherapy cares. Extended knowledge of possible adverse drug reactions may improve patient adherence to therapeutic regimen even in case of interactions that may be accepted, if not unknown. By strengthening the relationship between doctor and patient, reaching a real therapeutic alliance, clinicians could guarantee the safety of treatments and reduce possible malpractice claims related to drug–drug interactions. It is necessary, then, to facilitate and promote a constant conversation with the patient [326], as well as careful clinical and laboratory monitoring in order to quickly discover any adverse event. DDIs, as previously discussed, can also determine liver or kidney overload and, therefore, require careful monitoring to quickly detect any drug interaction. The timely identification of pathological effects of drug interactions allows the right therapeutic changes, thus avoiding potential organ damage and subsequent harm to the patient. The benefits of this strategy will not only be economic, allowing to reduce the costs of compensation and civil disputes, but will strengthen the patient’s trust in the doctor, thus enhancing his diagnostic and therapeutic role and making the therapeutic alliance more effective.

## 5. Conclusions

The management of peripheral vestibular vertigo in elderly requires a deep knowledge of clinical pharmacology and of multidisciplinary issues in the treatment of these patients. Pharmacogenomics are a key point of the therapeutic algorithm. Individual differences in ageing seem to be strictly related to genotype. The evaluation of the different alleles linked to CYP450 isoforms and transporters may be useful to choose the best therapeutic option [102,115,125,159,327]. Clinicians must always remember the important changes related to ageing, involving Vd, renal clearance, hepatic function, and multiple organ impairment. However, individual differences in concomitant treatment, sex, comorbidities, physician decisions, and lifestyle may add great variability to this process [5,254,256]. A good number of therapeutic options is available for peripheral vestibular vertigo. None of the mentioned drugs has shown a superiority in clinical trials; therefore, physician’s challenge consists in the choice of the proper compound [14,22,25,41,236,237,238,245]. The knowledge of DDIs can be used to select treatments for patients, thus optimizing clinical response, minimizing toxicity, and improving the safety of care. DDIs are potentially harmful to patients, so full information is an essential requirement also to enhance a therapeutic alliance and to avoid malpractice claims.

However, we need to underline that most of DDIs described do not show a clinically significant effect because in clinical practice other variables play a role (e.g., inter-individual differences, enzyme availability, concentration of drug into the site of metabolism, affinity of drugs for the enzyme [19,328,329,330,331,332], and activity of genetic and epigenetic factors [333,334]).

## Figures and Tables

**Table 1:** Rationale, current clinical indications, and dosages of peripheral vestibular vertigo drugs.

	Mechanism(s) of Action	Current Clinical Indications	Dosage Suggested	Route of Administration
Betahistine	Strong antagonist of histamine H3 receptors and a weak agonist of H1 receptors [22,23]	MD and other causes of peripheral vestibular vertigo [22,24,25]	24/48 mg daily [23]	OS [23]
**Diuretics**				
Acetazolamide	Inhibition of carbonic anhydrase [30]	MD [22]	250–375 mg daily [30]	OS [30]
Hydrochlorothiazide	Inhibition of sodium chloride co-transporter in the distal convoluted tubule [31,32]	MD [22]	12.5/25 mg daily (hypertension) [31]	OS [31]
Spironolactone	Mineralocorticoid receptor antagonist [33,34,35]	MD [22]	25–100 mg daily (hypertension) [33,34]	OS, IV [33,34]
Triamterene	Inhibition of ENaC [35]	MD [22]	50/100 mg daily [36], but may vary depending on formulation/coformulation [37,38]	OS [36]
**Benzodiazepines**				
Clonazepam	Allosteric modulation of GABA_A_ receptor [39,40]	All forms of PVV [41,42]	1.5–20 mg/die, generally 3–6 mg/die (OS) [43,44]	OS [40]
Diazepam	Allosteric modulation of GABA_A_ receptor [39,40]	All forms of PVV [41,42]	4–60 mg/daily (OS) 10–60 mg/daily (IV, IM) [45]	OS, IV, IM, rectal [40]
Lorazepam	Allosteric modulation of GABA_A_ receptor [39,40]	All forms of PVV [41,42]	2–10 mg/daily [44,46]	OS, IM, IV [40]
**Anticholinergics**				
Atropine	Non-selective muscarinic blocker [47]	All forms of PVV [22,25,41]	0.3–4 mg (depending on clinical indication) [47,48]	IV, IM, SC [47,48]
Glycopyrrolate	Non-selective muscarinic blocker [49]	All forms of PVV [22,25,41]	2 mg in clinical trial [50], but may vary depending on clinical indication 1–8 mg (OS) [51] Various (parenteral) [49,52]	IV, OS, IM [49,51,53]
Scopolamine	Non-selective muscarinic blocker [54]	All forms of PVV [22,25,41]	0,25–1 mg daily (IM, IV) [54]. 0.5 mg (TD) in clinical trial [55]	IM, IV, TD [54,55]
**Antihistamines**				
Dimenhydrinate + cinnarizine	D: antagonist of H1 receptor [4] C:It blocks voltage-gated calcium channels, preventing calcium translocations across the vestibular air cells and, thus, regulating hair cell afferent vestibular transmission, anti-vasoconstrictor activity, reduction in the blood viscosity of the inner ear’s circulatory system [4]	All forms of PVV [22,25,41,56]	Dimenhydrinate: 25–200 mg (OS) [57] Basis of 50 mg (IV-IM), but may vary [58] Cinnarizine: 15–225 mg (OS) [13,59]. Recommended clinical dose (vestibular disorders): varies between 25 mg thrice-daily and 75 mg once-daily, up to a maximum of 225 mg [13] Dimenhydrinate/ cinnarizine (co-formulation): 20/40 mg thrice a day [60]	IV, IM, OS (d) [57,58] OS (c) [59] OS (co-formulation) [60]
Diphenhydramine	Antagonist of H1 receptor [61,62]	All forms of PPV [22,25,41,56]	25 mg-50 mg [61,63,64]	OS [61,63]
Meclizine	Antagonist of H1 receptor [65]	All forms of PVV [22,25,41,56,66]	12.5–25 mg [67,68]	OS [67]
Promethazine	Antagonist of H1 receptor [69,70]	All forms of PVV [22,25,41,56]	25–100 mg (OS) 25–50 mg; max: 100 mg (IM, IV) [69] May vary [71]	OS, IM, IV [69]
**Other antiemetics**				
Metoclopramide	It acts on 5HT4 (agonist), 5HT3 (antagonist) and dopamine D_2_ (antagonist) receptors [40,71,72]	All forms of PVV [41,73]	10 mg–30 mg or max 0.5 mg/kg (IV-IM-OS) [72,74]	OS, IM, IV, rectal [72,74]
Ondansetron	5HT3 antagonist [75,76]	All forms of PVV [22,25,41,56]	4–8 mg capsules (OS), multiple administration also 8 mg (IV-IM) 16 mg (rectal) [76]	OS, IM IV, rectal [76]

ENaC, epithelial sodium channel; GABA, gamma aminobutyric acid; IM, intramuscular; IV, intravenous; MD, Meniere’s disease; OS, oral; PVV, peripheral vestibular vertigo; SC, subcutaneous; TD, transdermal.

**Table 2:** Pharmacokinetics of drugs used in peripheral vestibular vertigo.

	Oral Bioavailability	Time to Peak Concentration	Serum Half-Life (t1/2)	Protein Binding	Transporter Proteins	Metabolism	Metabolites
BHS	NA	1 h	3.5 h	5%	-	Monoamine oxidases (MAO) A/B	2-pyridylacetic acid (2-PAA)
**Diuretics**							
AAZ	NA	2 h	8 h	90–95%	-	-	-
HCT	70%	4 h	6–25 h	40–70%	-	Mainly unmodified	2-Amino-4-chlorobenzenesulfonamide
SPL	~65%	2–4 h * (including metabolites)	1.5 h *	90% *	-	First pass metabolism	Canrenone, 6-ß-hydroxy-7-α-(thiomethyl) spironolactone, 7-α-(thiomethyl) spironolactone (TMS),
TRI	52%	3 h	2–4 h	67%	-	Liver	Hydroxytriamterene
**Benzodiazepines**							
CLZ	90%	1.2 h	23 ± 5 h	82–86%	-	Liver (glucuronidation), CYP3A4	7-aminoclonazepam and 7-acetamido-clonazepam
DZP	90–100%	0.5–1.5 h	24–48 h	96–98%	-	Liver (glucuronidation), CYP3A4, CYP2C19	Desmethyldiazepam, oxazepam, temazepam
LOR	90%	2–3 h	12–16 h	85–90%	-	Liver (glucuronidation)	3-O-phenolic glucuronide
**Anticholinergic drugs**							
ATP	-	10 min (IM)	4 h	-	-	50% liver 50% unmodified	NA
GLY	3% (children) NA, but higher (adults)	NA	0.83 ± 0.27 h (IV) 75 min (IM) 2.5–4 h (OS, solution)	-	-	NA	NA
SCO	NA	2 min (IM) 24 h (TD)	8 h	-	-	Hepatic	NA

* Protein binding is about 98% for canrenone; canrenone half-life = 9–24 h; time to peak concentration of spironolactone alone = 2.6 h. AAZ; acetazolamide; ATP, atropine; BHS, betahistine; CLZ, clonazepam; CYP, cytochromes P450; DZP, diazepam; GLY, glycopyrrolate; HCT, hydro-chlorothiazide; IM, intramuscular; IV, intravenous; LOR, lorazepam; MAO, monoamine oxidase; NA, not available; OS, oral; P-gp, p-glycoprotein; SCO, scopolamine; SPL, spironolactone; TD, transdermal; TRI, triamterene.

**Table 3:** Pharmacokinetics of antihistamines and other antiemetics.

	Oral Bioavailability	Time to Peak Concentration	Serum Half-Life (t1/2)	Protein Binding	Transporter Proteins	Metabolism	Metabolites
**Antihistamines** **(H1 Antagonists**)							
DIM + CNZ	43–72% (d)	1–4 h (d) 2–4 h (c)	6–7 h (d) 4–5 h (c)	80–85% (d)	-	Hepatic (d, *see the section below*). CYP2D6 and CYP2B6, but other CYP may be involved (c)	D: Diphenhydramine, DMDP C: Conjugated with glucuronic acid
DPH	43–72%	1–4 h	3–9.3 h	80–85%	-	Hepatic first-pass metabolism CYP2D6, and to a minor extent CYP1A2, CYP2C9 and CYP2C19	DMDP
MEC	NA	1.5–6 h	5.21 ± 0.80 h	NA	-	Hepatic CYP2D6	Norchlorcyclizine (rats), 10 different metabolites in human urines. Human metabolites have not been identified, but meclizine undergoes aromatic hydroxylation or benzylic oxidation.
PMZ	25%	2–3 h	4–6 h (OS) 9–16 (IV) 6–13 (IM)	-	-	Hepatic first-pass metabolism	Promethazine sulfoxide (PMZSO), N-demethylpromethazine
**Other antiemetics**							
MCP	35–100%	0.5–2 h (OS) 3 h (IM)	5–6 h	13–40%	-	Hepatic: CYP2D6 isoform, and possibly CYP1A2 (minor role), CYP2C19and CYP3A; conjugation.	Argikar et al. identified 10 metabolites of metoclopramide (M1-M10) in the urine after oral administration. Of those (M1, M2, M6, M7, and M8) were conjugated to either glucuronide or sulfate. Mono-de-ethyl-metoclopramide and N-4 sulphate conjugated are two important products.
OND	56% (OS) 60% (rectal)	1.5 h (OS) 6 h (rectal)	3–6 h	70–76%	P-gp substrate	Hepatic first-pass metabolism, CYP1A2, CYP2D6, CYP3A4	Hydroxylation on the indole ring followed by subsequent glucuronide or sulphate conjugation.

CNZ, cinnarizine; CYP, cytochromes P450; DIM, dimenhydrinate; DMDP, monodesmethyldi-phenhydramine; DPH, diphenhydramine; IM, intramuscular; IV, intravenous; MCP, metoclopramide; MEC, meclizine; NA, not available; OND, ondansetron; OS, oral; P-gp, p-glycoprotein; PMZ, promethazine; P-gp, p-glycoprotein; TD, transdermal.

**Table 4:** Dose changes, elimination, and inductor/inhibitor activity of peripheral vestibular vertigo drugs.

	Enzymes Inductor/Inhibitor	Elimination	Dose Changes in Hepatic Disease	Dose Changes in Renal Disease	References
BHS	-	85% urine Low levels in bile	No dosage adjustment seems to be needed	No dosage adjustment seems to be needed	[23,29]
**Diuretics**					
AAZ	-	100% urine	Contraindicated in hepatic impairment	Dosage adjustment needed. Contraindicated in patients with IR but may be used in cardiopathic patients without severe renal impairment	[30,77]
HCT	-	60–80% urine 24% feces	Caution needed in patients with severe hepatic impairment	The compound is not effective in patients with eGFR <30 mL/min Do not administer in patients with severe renal impairment	[31,78]
SPL	-	Mainly in urine, secondary in bile	Caution needed in patients with severe hepatic impairment	Avoid in acute kidney injury, anuria or eGFR <30 mL/min. Pay attention in case of renal impairment	[33,34,40]
TRI	-	The majority is expelled in urine, a minor quote in bile	Caution needed in patients with impaired hepatic function. In patients with severe hepatic impairment, triamterene’s levels may increase. In NIH data bank it is contraindicates in severe hepatic impairment	Caution needed. Periodic BUN and serum potassium determinations should be made to check kidney function, especially in patients with suspected or confirmed renal insufficiency	[36,37,40,79,80]
**Benzodiazepines**					
CLZ	-	50–70% in urine 10–30% feces	Protein binding may be changed by cirrhosis, increasing the free fraction. Caution needed. Contraindicated in severe hepatic impairment	Caution needed	[40,43,44,81]
DZP	-	100% urine	Contraindicated in severe hepatic impairment. Caution needed in other mild and moderate hepatic impairment	Caution needed	[40,44,45,82]
LOR	-	88 ± 4% urine 7 ± 2% feces.	Caution needed. Contraindicated in severe hepatic impairment	Caution needed in severe hepatic impairment	[44,46,83,84]
**Anticholinergic drugs**					
ATP	-	50% liver50% urine	Caution needed	Caution needed	[47,48,85]
GLY	-	Urine, only 5% bile	Further studies needed. Since kidney elimination has a major role, hepatic impairment seems not to be relevant, despite a certain negative effect of anticholinergic drugs on hepatic damage	Dose reduction by 30% in patients with mild to moderate renal impairment. Contraindicated in severe renal impairment	[49,52,86]
SCO	-	Urine	Caution needed for the risk of CNS reactions	Caution needed for the risk of CNS reactions	[54,85,87]
**AHs (H1-antagonists)**					
DIM + CNZ	Inhibition of CYP2D6 (d)	Mainly in urine (d) 40–60% feces and minor quote in urines (c)	Caution needed (d) Coadministration contraindicated in patients with severe hepatic impairment	Caution needed (d) Coadministration contraindicated in patients with eGFR < 25 mL/min	[57,60,61,71,88,89]
DPH	It inhibits CYP2D6	Mainly in urine	Caution needed	Caution needed	[61,71,90,91,92]
MEC	Meclizine seems to reduce the expression of CYP2B10, 3A11, 1A2 in experimental models	Urine, feces	Caution needed (need further evaluation)	Caution needed (need further evaluation)	[65,67,93,94]
PMZ	-	Urine	Caution needed (need further evaluation)	Caution needed (need further evaluation)	[69,70,71,95]
**Other antiemetics**					
MCP	-	86% urine, minor quote in bile	Caution needed	Caution needed In patients with last stage renal impairment (eGFR ≤ 15 mL/min): Dose reduction of 75%. Severe/moderate renal impairment (eGFR 15–60 mL/min): Dose reduction of 50%	[71,72,74,96,97,98]
OND	-	Majority hepatic, 5% urine	Caution needed, especially in severe hepatic impairment	Caution needed, although studies on moderate renal impairment did not show significant changes	[40,75,76,99,100]

AAZ, acetazolamide; ACDs, anticholinergic drugs; AHs, antihistamines; ALT, Alanine Aminotransferase; ATP, atropine; BDZ, benzodiazepines; BHS, betahistine; BUN, Blood Urea Nitrogen; CLZ, clonazepam; CNS, central nervous system; CNZ, cinnarizine; CYP, cytochromes P450; DIM, dimenhydrinate; DPH, diphenhydramine; DZP, diazepam; eGFR, estimated glomerular filtration rate; GLY, glycopyrrolate; HCT, hydrochlorothiazide; LOR, lorazepam; MCP, metoclopramide; MEC, meclizine; NIH, National Institutes of Health; NA, not available; OND, ondansetron; P-gp, P-glycoprotein; SCO, scopolamine; PMZ, promethazine; SPL, spironolactone; TRI, triamterene.

**Table 5:** Peripheral vestibular vertigo drugs pharmacokinetic interactions.

	Effect of the Combination	Mechanism of Interaction	Selection of Drugs Affected	Clinical Comment
**Betahistine**	Serum level ↑ by MAOIs [23]	Inhibition of MAOIs	-	Decrease the dosage
CYP1A2 substrates	Serum level may be slightly increased by amiodarone, ciprofloxacin, fluvoxamine, fluoxetine, antifungals, duloxetine, isoniazid [101,102,103,104]	Inhibition of CYP1A2	Diphenhydramine (minor extent) [92]	Reduce the dosage
	Serum level slightly decreased by tocilizumab, rifampicin, antiseizure drugs, smoke [104,105]	Induction of CYP1A2		
CYP2C19 substrates	Serum level ↑ by inhibitors: voriconazole, fluconazole, omeprazole, esomeprazole, fluvoxamine, fluoxetine, cimetidine, modafinil, felbamate, armodafinil, ticlopidine [45,106,107,108,109]	Inhibition of CYP2C19	Diazepam, diphenhydramine (minor extent) [45,92]	Reduce the dosage (of 5 mg) Omeprazole may increase diazepam levels, associated to dementia risk. PPI inhibitors are also related to dementia [110]
	Serum level ↓ by inducers: rifampicin, enzalutamide, efavirenz, ritonavir, tocilizumab [45,105,106,109]	Induction of CYP2C19		Evaluate dose increase
CYP2C9 substrates	Serum level ↑ by amiodarone, some antifungals, fluoxetine, fluvoxamine, valproic acid, disulfiram, fluvastatin [101,109,111,112]	CYP2C9 inhibition	Diphenhydramine (minor extent) [92]	Low grade of interaction
	Serum level ↓ by tocilizumab, antiseizure drugs, rifampicin, enzalutamide, ritonavir, aprepitant [105,109]	CYP2C9 induction		
CYP2D6 substrates	Serum level ↑ by amiodarone, abiraterone, other anticancer drugs, bupropion, celecoxib, HCQ, labetalol, vemurafenib some antipsychotics, some antidepressants [101,105,109,113,114,115,116,117,118,119]	CYP2D6 inhibition	Diphenhydramine, meclizine, metoclopramide [92,94,97]	Dose decrease Meclizine (from 25 mg to 12.5 mg)
CYP3A4 substrates	Serum level ↑ by inhibitors: some antifungals omeprazole, esomeprazole, fluvoxamine, cimetidine, isoniazid, cimetidine, idelasilib, antidepressants, calcium- antagonists, amiodarone, erythromycin, crizotinib, imatinib other anticancer drugs, calcineurin inhibitors, mTOR inhibitors, clarithromycin, erythromycin and troleandomycin, remdesivir, tofacitinib, some antidepressants [43,45,101,102,103,105,106,107,108,109,117,118,120,121,122,123,124,125,126,127,128,129,130]	Inhibition of CYP3A4	Diazepam, clonazepam [43,45], corticosteroids [131,132,133,134]	Dose decrease (Diazepam of 5 mg, Clonazepam, and corticosteroids of one half) Antidepressants don’t affect clonazepam levels [43]
	Serum level ↓ by inducers: modafinil, armodafinil, carbamazepine and other anti-seizure drugs, rifampicin, tocilizumab [45,105,106]	Induction of CYP3A4		Evaluate dose increase
Diazepam	Serum level ↑ by disulfiram [45]	Inhibition of oxidative metabolism [135]	-	Evaluate dose reduction
	Serum level ↑ by ciprofloxacin [106]	CYP1A2 inhibition (even if diazepam is not metabolized by this isoform) or unknown mechanism of reduced clearance	-	Evaluate dose reduction
	Serum levels ↓ by food, antiacids, narcotics [45]	Reduction of diazepam absorption	-	Dose increase related to patient condition. Food and antiacids reduce concentration peak, but not AUC
	Serum levels ↑ by prokinetics [45]		-	Evaluate dose reduction
**Lorazepam**	Serum levels ↑ by valproate and probenecid	Probenecid decreases lorazepam clearance, reducing the production of lorazepam-glucuronide. Similar mechanism for valproate [46,106]	-	Dose decrease
P-gp substrates	Serum level ↑ by CQ, HCQ, LPV/r, canagliflozin, lansoprazole, tamoxifen [136], some antipsychotics, some calcium antagonists, calcineurin inhibitors, lovastatin [101,103,105,109,117,119,120,121,122,123,124,129,130,133,137,138,139,140,141,142,143,144,145,146]	P-gp inhibition	Ondansetron, dexamethasone [100,147]	Ondansetron may have a pronociceptive effect with P-gp inhibitors [100], therefore evaluate a dose decrease, even if ondansetron is used for few days (maximum of 5) [76] Dexamethasone or P-gp inhibitor reduction may be needed
	Serum level ↓ by dexamethasone [147]	P-gp induction	Ondansetron, dexamethasone [100,147]	Risk for reduced efficacy

AUC, area under the curve; CQ, chloroquine; CYP, cytochrome P450; HCQ, hydroxychloroquine; LPV/r, lopinavir/ritonavir; MAOIs, monoamineoxidaseinhibitors; mTOR, mechanistic target of rapamycin; P-gp, p-glycoprotein.

**Table 6:** Peripheral vestibular vertigo drugs pharmacokinetic interactions (perpetrators).

	Effect of the Combination	Mechanism of Interaction	Selection of Drugs Affected	Clinical Comment
AAZ	↑ Phenytoin and carbamazepine concentration. ↓ Primidone levels	It acts on the cited anti-seizure drugs absorption or has an unknown mechanism [30,148,149]	-	Risk for increased or decreased efficacy
	↑ Tricyclic antidepressants and amphetamine levels. ↓ Lithium and aspirin levels	It facilitates/lasts the elimination of these substances [30,150]	-	Risk for adverse events or decreased efficacy
	↑ Lithium levels	It reduces lithium urinary excretion [31,151,152]	-	Risk of increased lithium side effects (see the spironolactone section)
CLZ	Variation of phenytoin levels [43]	Unknown. Probably clonazepam stimulate inhibition of enzymatic system in some patients, and induction in others [153]	-	Risk for adverse events or decreased efficacy
	↑ Primidone levels [43,154]	Unknown pharmacokinetic mechanism [154]	-	Risk for adverse events
CYP3A4 substrates	Dexamethasone ↓ substrates levels [147]	Induction of CYP3A4	Gliquidone, glyburide, repaglinide, pioglitazone, saxagliptin, warfarin, antiplatelet drugs, some antiarrhythmics, calcium antagonists, direct oral anticoagulants, some statins, some NSAIDs, some opioids, some β2-agonists, PPI (minor extent), anticancer drugs (e.g., cyclophosphamide, docetaxel, etoposide, gefitinib), calcineurin and mTOR inhibitors, macrolides (e.g., clarithromycin and telithromycin), remdesivir, HCQ, some JAK inhibitors, some antipsychotics, some antidepressants [101,102,103,105,107,108,113,114,115,116,117,119,120,121,122,123,124,125,126,127,128,138,139,140,155,156,157,158,159,160,161,162,163,164,165]	CYP3A4 induction accelerates the production of antiplatelets active metabolites [101,102,103] Pravastatin is not a relevant substrate of CYP450 and then may be a safer choice [101] The interaction may accelerate the production of metabolites or delay opioids’ clearance [166]
CYP2D6 substrates	Serum level ↑by diphenhydramine and dimenhydrinate	CYP2D6 inhibition	Beta-blockers (e.g., metoprolol, bisoprolol, carvedilol), calcium antagonists, antidepressants, antipsychotics, codeine, hydrocodone, antiarrhythmic drugs (often class I), formoterol, anticancer drugs(e.g., tamoxifen, gefitinib), remdesivir, HCQ, upadacitinib, some antipsychotics, some antidepressants [101,102,103,105,119,155,159,160,161,163,164,165,166]	Risk of adverse events Interactions may stop the production of metabolites or opioids’ clearance [166]
DPH	Barbiturates, sulfacetamide, sodium salicylate	It may reduce the absorption of these drugs [61]	-	Dosage increase may be needed
HCT	↑ lithium levels [31,151,152]	Reduction of lithium clearance	-	Risk for ADRs. It is important to maintain lithium in the optimal range [167], therefore clinician should evaluate dose reduction for spironolactone or therapeutic switch. However, lithium itself may be switched to use other mood stabilizers [168]
MCP	Digoxin	Metoclopramide decrease digoxin absorption acting on gut motility [72,169]	-	Decrease of metoclopramide dosage, monitoring digoxin plasmatic range
	Ciclosporin	Increase of ciclosporin absorption. Metoclopramide hastens gastric emptying and facilitates small intestine absorption [170]	-	Dosage reduction of metoclopramide/ ciclosporin may be needed
	Mivacurium, succinylcholine	It may reduce their clearance blocking plasmatic cholinesterase [72]	-	Dosage reduction of metoclopramide may be needed
P-gp substrates	Dexamethasone ↓ substrates levels [147]	P-gp induction by dexamethasone	DPP-4 inhibitors, SGLT-2 inhibitors, DOAC, verapamil, aliskiren, digoxin, amiodarone, atorvastatin and simvastatin, some β2-agonists, some anticancer drugs, calcineurin and mTOR inhibitors, Fluoroquinolones, daptomycin, linezolid, baricitinib, dexamethasone, remdesivir, upadacitinib (in vitro) [101,102,103,105,117,120,121,122,123,124,125,126,127,138,139,140,144,145,146,155,156,157,158,163,171,172]	Increase dosage of P-gp substrates/decrease dexamethasone depending on clinical control of hypertension, diabetes, or other pathologies and on temporal duration of corticosteroid treatment
SPL	↑ Lithium levels	Reduction of lithium clearance	-	Risk for adverse events. It is important to maintain lithium in the optimal range [167], therefore clinician should evaluate dose reduction for spironolactone or therapeutic switch. However, lithium itself may be switched to use other mood stabilizers [168]
	↑ Digoxin level	Reduction of digoxin clearance [173,174]	-	Risk for adverse events. Evaluate dose reduction to maintain digoxin in its therapeutic range [175]
TRI	↑ Lithium levels may increase	Reduction of lithium clearance	-	Risk for increased adverse events (see the spironolactone section)

AAZ, acetazolamide; CLZ, clonazepam; CYP, cytochrome P450; DOAC, direct-acting oral anticoagulants; DPH, diphenhydramine; DPP-4, dipeptidyl peptidase-4; HCQ, hydroxychloroquine; JAK, Janus kinase; mTOR, mechanistic target of rapamycin; MCP, metoclopramide; NSAID, Nonsteroidal anti-inflammatory drugs; P-gp, p-glycoprotein; PPI, proton pump inhibitors; SGLT-2, sodium glucose co-transporter-2; SPL, spironolactone; TRI, triamterene.

**Table 7:** Pharmacodynamic drug interactions.

	Drug(s) Involved	Mechanism of Interaction	Clinical Comment
BHS	Antihistamines	Betahistine acts as an histamine analogue, with possible interactions with antihistamines as a consequence [23]	Risk for reduced efficacy
	Olanzapine	Coadministration with olanzapine showed a reduction of olanzapine binding to D_2_R	This may prevent adverse events (weight gain) related to olanzapine, but also “dopaminergic supersensitivity” due to high stimulation on D_2_R. Despite this evidence, betahistine may also affect drugs effect on D_2_R [176]
**Diuretics**			
Class effects	NSAIDs	Various mechanisms including NSAID related sodium and water retention, suppression of plasma renin activity, alterations in adrenoceptor sensitivity and impaired synthesis of vasodilator prostaglandins. Nephrotoxic effect and inhibition of natriuretic response to diuretic should be considered [31,36,40,80,177]	Risk for reduced efficacy of diuretics and kidney injury
	Other diuretics	Diuretic therapy combined with other antihypertensive drugs may lower blood pressure [40,41,178]	In case of multidrug therapy in a patient with MD and hypertension, the combination of two or more drugs acting on blood pressure should be evaluated carefully [34,36,150]. Clinicians may evaluate adding or replacing of one or more drugs used for hypertension
	Drugs prolonging QT, proarrhythmic drugs	Hypocalcemia, hypokalemia, and hypomagnesemia may also prolong QT interval [179], so diuretic therapy (associated with electrolytic disturbances) may worsen this side effect [180,181]	Caution needed. Switching to a drug which doesn’t alter QT, may be a good choice
HCT	Drugs which lower potassium (e.g., amphotericin B, corticosteroids, diuretics, laxatives, digitalis) and sodium levels (e.g., desmopressin, antidepressants, diuretics, carbamazepine)	The thiazide diuretic lowers potassium and sodium concentration [31]	Risk of hypokalemia and hyponatremia
	Antiarrhythmic drugs (e.g., amiodarone), antipsychotics (e.g., chlorpromazine) antimalarial drugs (e.g., chinidine)	Hypokalemia may facilitate torsade de points [31]	Risk of torsade de points
	Antidiabetics or drugs raising glucose levels	Increase of glucose levels [31,182]	Risk of hyperglycemia or hypoglycemia
	Calcineurin inhibitors, corticosteroids, mycophenolate, azathioprine and (lower risk) mTOR inhibitors	Immunosuppression and kidney transplant have a certain association with non-melanoma skin cancer: Thiazides, used in the management of MD, are associated with a certain skin cancer risk [183]	mTOR inhibitors (sirolimus and everolimus) have a lower risk, partially reverting oncogenic effects [184]. Avoid the combination is the optimal choice. Sun protection has an important role
SPL	Drugs generating hyperkaliemia (e.g., trimethoprim, ARB, ACE inhibitors, other potassium sparing diuretics, heparin, NSAIDs, ciclosporin, tacrolimus)	Spironolactone may increase potassium levels [33,34]	Risk for hyperkaliemia
	Cholestyramine	Cholestyramine increases the urinary excretion of bicarbonate and spironolactone favors acidosis with potassium reabsorption and reduction of urine acidity [185]	Risk of hyperkalemic metabolic acidosis
	Antipsychotics and metoclopramide	Hyperprolactinemia is a possible side effect of antipsychotics, related to their inhibition on D_2_R. This condition causes several clinical manifestations including menstrual disturbances, sexual dysfunction, galactorrhea, gynecomastia, infertility [186]. Spironolactone may cause gynecomastia or sexual disturbances [34]. Metoclopramide is also associated to hyperprolactinemia and, moreover, to depression [97]	Hyperprolactinemia related to antipsychotics seems to be more frequent in females and to be mainly connected with some specific compounds (risperidone, haloperidol, amisulpride) [187]. Spironolactone should be avoided in male patients in treatment with prolactin raising antipsychotics or prolactinoma [188]
TRI	Skeletal muscle relaxants (non-depolarizing) and preanesthetic/anesthetic agents	Triamterene enhances the effect of the cited drugs [36]	Risk for adverse events
	ACE inhibitors, angiotensin receptor blockers (ARB) or potassium containing medication	Triamterene causes the raise of potassium levels	Risk of hyperkaliemia [36]
	Antidiabetic drugs, or drugs increasing glucose levels	It may also raise blood glucose levels [36,37,80]. The reason for this mechanism seems to rely in triamterene’s inhibition of G-protein-coupled bile acid receptor 1 (GPBAR1, also known as TGR5) [189,190]	Risk of hyperglycemia or hypoglycemia
**BDZ**			
Class effects	Xanthines contrast BDZ anxiolytic effect [45]	Xanthines may give anxiety [191]	Reduced clinical effect
	CNS depressants (opioids, antipsychotics, antidepressants, hypnotics, antiseizure drugs, anesthetics, antihistamines)	When associated with other central nervous system (CNS) depressants BDZ effect may be enhanced [43,45,46,192]	Risk benefit-management is needed
	Aminophylline and theophylline can reverse at least partially the sedation from BDZ	This interaction appears to be due to the blockade of adenosine receptors by aminophylline [106]	Risk for reduced efficacy
DZP	Dopamine levels down when administered with L-DOPA [45,193]	Benzodiazepines influence dopamine release [194]	Difficult management of Parkinson’s disease. Coadministration of antipsychotics in this clinical setting may worsen clinical status, increasing extrapyramidal side effects [193]
LOR	Clozapine: concomitant use of lorazepam may produce marked sedation, excessive salivation, hypotension, ataxia, delirium, and respiratory arrest [84,195]	Unknown	Evaluate the real necessity of this combination, switching eventually to other compounds
**ACDs**			
Class effects	KCL	Anticholinergics reduce gastric transit and KCL has an irritating effect [47,196]	Risk of gastric bleeding
	Other drugs with anticholinergic activity (e.g., antipsychotics, amantadine, tricyclic antidepressants, nefopam, MAO inhibitors, anti-Parkinson drugs) may worsen side effects	Concomitant action on ACh pathway [47,49,54]	Increased adverse events
	AChE inhibitors, cholinergic drugs	Concomitant administration of AChE inhibitors and anticholinergics must be avoided in patients with QT prolongation [47,49,54]. The concomitant consumption of cholinergic drugs is another contraindication to the use of anticholinergics [54]	Risk of arrythmias, pharmacodynamic antagonism
	Antihypertensive/hypertensive drugs	Anticholinergic drugs may increase (or lower) blood pressure and raise heart rate [54,197]	Evaluate the real utility of coadministration and monitor patient
	PPI	Chronic use of PPI may be associated with dementia, and this risk should be added to that generated using [110,198]	Avoid chronic treatment with PPI and ACD
	Tricyclic antidepressants	These drugs act also on histaminergic and cholinergic receptors with antagonist effect [40,199] and for this reason they may enforce side effects related to antihistamines and anticholinergics	Avoid coadministration
	Antipsychotics	Some anticholinergics drugs are used in the treatment of Parkinson’s disease, whereas other are associated with parkinsonism. Antipsychotics generate extrapyramidal effects [54,193,200,201]	Avoid coadministration
	Opioids	Opioids and anticholinergics both lead to constipation [202,203]	Avoid coadministration
**Antihistamines**			
Class effects	Anticholinergics	It has anticholinergic effects so it must be avoided in BPH, glaucoma, myasthenia gravis, bradycardia, gastrointestinal paralysis, or obstructions [57,61,67,69]	Avoid coadministration
	CNS suppressant drugs (e.g., MAOIs) [57,61,67,69].	Sedation is a common adverse event of antihistamines [192]	Avoid coadministration
	Tricyclic antidepressants	These drugs act also on histaminergic and cholinergic receptors with antagonist effect [40,199] and for this reason they may enforce side effects related to antihistamines and anticholinergics	Avoid coadministration
DIM	Ototoxic drugs	When administered with ototoxic drugs, dimenhydrinate may hide ototoxicity [57]	Caution needed
	Drugs prolonging QT, proarrhythmic drugs	Diphenhydramine (and then dimenhydrinate) may also prolong QT, but rarely [204,205]	Avoid coadministration
DPH	Antihypertensive drugs	It may cause fatigue, that is a possible adverse event of antihypertensive drugs also [61,206]	Prefer a short time of treatment
	Drugs prolonging QT, proarrhythmic drugs	Diphenhydramine (and then dimenhydrinate) may also prolong QT, but rarely [204,205]	Avoid coadministration
PMZ	Epinephrine	Promethazine may reverse epinephrine’s vasopressor effect [70]	Epinephrine should not be used to treat hypotension associated with promethazine overdose
**Other drugs**			
OND	Drugs prolonging QT interval (e.g., antiarrhythmics, antipsychotics, antidepressant, antihistamines, some antimicrobial, methadone, HCQ), cardio-toxic, pro-arrhythmic drugs	Ondansetron may prolong QT interval and should be administered carefully in cardiologic setting and in people with Long QT Syndrome (LQTS) [76]	Avoid the coadministration. Consider patient risk factors is also important. Freedman et al. showed that most cases were related to intravenous ondansetron, especially if administered with other drugs prolonging QT. Therefore, they suggest ECG and electrolyte screening only in patients at high risk and receiving ondansetron intravenously [205,207]
	Tramadol, SSRI, SNRI, serotoninergic drugs	Tramadol acts on serotonin reuptake, whereas ondansetron is a 5HT_3_ receptor antagonist. Other authors suggest also a pharmacokinetic mechanism: competition at CYP2D6 leading to the reduction of tramadol active metabolite [208]	Ondansetron may reduce tramadol’s analgesic effect and increase the risk of serotoninergic syndrome [76,208]
**MCP**	L-DOPA, dopamine agonists	Metoclopramide is an antagonist of dopamine D_2_ receptors [40,71,72]	Avoid coadministration
	Alcohol and CNS depressants	Increase of sedative action [72,192]	Avoid coadministration
	Anticholinergics and morphine derivatives	These drugs may antagonize the gastrointestinal effect of metoclopramide (a pro-kinetic drug). They are also related to constipation. However, this last effect is useful to avoid diarrhea from MCP [202,203]	Avoid coadministration of anticholinergics and MCP for the best clinical effect. Evaluate risk benefit or the use of another compound if patient consumes opioids for pain management
	Psychiatric drugs	Antipsychotics may increase the risk of extrapyramidal side effects and antidepressants or serotoninergic drugs (e.g., SSRI, SNRI) may increase the risk of serotoninergic syndrome, since metoclopramide acts on dopamine and serotonin receptor [72]	Avoid coadministration
	Antihypertensive, hypertensive drugs	Metoclopramide leads to hypotension [209]	Administer carefully. Metoclopramide is administered for a short interval of time (5 days) [72]. Therefore, clinician should adequate antihypertensive therapy, reducing metoclopramide and/or antihypertensive drug dosage
CCS	Antidiabetics or drugs increasing glucose levels	Glucocorticoids are associated to the raise of glucose levels [210]	Avoid the coadministration
	NSAIDs, PPI	Risk of bleeding related to CCS [211], creating antagonism with PPI	CCS may be dangerous, especially if patient is affected by gastrointestinal ulceration (bleeding risk). The situation may be even more complex in the case patient consumes NSAIDs for pain treatment [211]
	Antipsychotics, antidepressants	Psychiatric drugs and CCS are often associated to weight gain [212]. CCS can induce psychiatric symptoms, worsening patients conditions [213]	Evaluate risk/benefit. Low-dose corticosteroid is not expected to generate psychiatric symptoms, whereas wait gain is a not desirable adverse event, which may happen in coadministration

ACDs, anticholinergic drugs; ACE, angiotensin-converting enzyme; ACh, acetylcholine; AH, antihistamines; ARB, angiotensin II receptor blockers: BDZ, benzodiazepines; BHS, betahistine; BPH, Benign prostatic hyperplasia; CCS, corticosteroids; CNS, central nervous system; CYP, cytochrome P 450; DIM, dimenhydrinate; DPH, diphenhydramine; DZP, diazepam;D_2_R, dopamine receptor D2; ECG, electrocardiogram; HCT, hydrochlorothiazide; KCL, potassium chloride; L-DOPA, levodopa; LOR, lorazepam; MAO, monoaminoxidase; MAOI, monoaminoxidase inhibitors; MCP, metoclopramide; MD, Meniere’s disease; mTOR, mechanistic target of rapamycin; NSAIDs, nonsteroidal anti-inflammatory drugs; OND, ondansetron; P-gp, p-glycoprotein; PMZ, promethazine; PPI, proton pump inhibitors; SNRI, serotonin-norepinephrine reuptake inhibitor; SPL, spironolactone; SSRI, serotonin reuptake inhibitors; TRI, triamterene.

**Table 8:** Adverse drug reactions related to the most common drugs used in the management of peripheral vestibular vertigo.

Anticholinergics	Most common adverse events include stypsis, dyspepsia, blurred vision, dry mouth, cognitive decline, urinary retention, flushing, tachycardia, hypertension (but also hypotension), and mydriasis [40,47,49,53,54,214]. Psychiatric, ocular, cutaneous, vascular, nervous symptoms, and dryness of the respiratory mucous membranes of the nasopharynx have also been reported [47,49,53,54,214]
Antihistamines	Sedation, CNS effects (e.g., vertigo, tinnitus, headache, blurred vision, diplopia, insomnia, tremor, fatigue), gastrointestinal symptoms, anti-muscarinic adverse events (e.g., urinary retention, dry mouth), allergic dermatitis, respiratory symptoms (related to the reduction of respiratory secretions) are common adverse events. Hematologic alterations, fever, cardiovascular symptoms, extrapyramidal symptoms (promethazine), jaundice are also described. Cinnarizine (administered with dimenhydrinate) is associated with CNS effects (e.g., extrapyramidal symptoms), somnolence, gastrointestinal adverse events, hypersensitivity, hematologic alterations [40,57,61,67,69].
Betahistine	Gastrointestinal symptoms and headache are the most common; cutaneous reactions, hypersensitivity, palpitation, feeling hot, eye irritation were also reported [23,27].
**Diuretics**	
Acetazolamide	Acidosis, electrolytic alterations (lowering of potassium and sodium levels), paresthesia, anorexia, tinnitus, gastrointestinal symptoms, polyuria, somnolence, confusion, altered taste. Suicide, cutaneous reactions, alteration of glucose levels, hepatic damage, hematopoietic alterations, other neurologic symptoms, nephrolithiasis and other reactions related to kidney damage were also reported [30,77].
Hydrochlorothiazide	Hypercalcemia, impaired glucose tolerance, increase of cholesterol and triglycerides, skin cancer, closed angle glaucoma, hypersensitivity, thrombocytopenia, hypomagnesemia, hypokalemia, hyponatremia, hypotension, gastrointestinal symptoms, cutaneous manifestations, impotence and others [31,182,183]. It may also cause exacerbations in patients with systemic lupus erythematosus (SLE) [31]. Despite this, hydrochlorothiazide showed a better safety than other thiazides [215].
Spironolactone	Hyperkaliemia, hyponatremia, hypovolemia, metabolic acidosis, gastrointestinal symptoms, sexual or hormonal changes (e.g., gynecomastia, erectile disfunction, amenorrhea), vocal alterations, kidney damage and urologic adverse events, hepatitis, cutaneous reactions, neurologic symptoms (e.g., lethargy, vertigo, ataxia; mainly in patients with hepatic encephalopathy), and leukopenia/thrombocytopenia are possible adverse events [34]. Gynecomastia is the most common collateral effect. In fact, spironolactone lacks of specificity for mineralocorticoid receptors and acts also on androgen and progesterone receptors [33,216].
Triamterene	Most frequent adverse events are hyperkalemia and gastrointestinal symptoms. Cutaneous reactions (including photosensitivity), dry mouth, megaloblastic anemia, pancytopenia, renal impairment and urinary symptoms, headache, and hyperuricemia were described [36,80].
**Other antiemetics**	
Metoclopramide	Metoclopramide’s more frequent adverse events are extrapyramidal effects, parkinsonism, galactorrhea, asthenia, somnolence, diarrhea, depression, and hypotension (intravenous administration) [72].
Ondansetron	Ondansetron’s main adverse events are: headache, fatigue, flushing, gastrointestinal symptoms, and cutaneous reactions in the site of injection [75,76].

CNS, central nervous system; SLE, systemic lupus erythematosus.

**Table 9:** Pharmacogenomics of vertigo drugs.

Drug Name	Protein Involved	Polymorphisms	Dose Adjustment
**Benzodiazepines**			
Class related Elements	GABA_A_ receptor	19 subunits of GABA_A_ receptor are described in literature: α (1–6), β (1–3), γ (1–3), δ, ε, π, θ, ρ (1–3). Properties of the assembled receptor will depend on nature of subunits. For example, DZP acts on receptor having α 1/2/3/5 subunits, but not on those expressing α4/6. For further details, Korpi and Sinkkonen wrote on this topic [298]	Unknown clinical implications. The process may lead to development of new benzodiazepines or selective use of available drugs
CLZ	CYP3A4	CYP3A4 1*B has a doubtful effect (see DZP section) [299]	Toth et al. showed a role of CYP3A4 different expression in dosage adjustment, but CYP expression was not different between people expressing different polymorphisms (no relation genotype-phenotype). However, the evaluation of low and normal expressers may be useful to dispose dose reduction (half- dose in low expressers) [299]
		CYP3A4*22 resulted in decreased activity [299]	See the section above. See DZP section for other possible polymorphisms of CYP3A4
	NAT2	NAT2*4 is associated with rapid acetylation of 7-amino-clonazepam (main metabolite), whereas NAT2*5, *6 and *7 are related to slow acetylation [299]	Slow acetylation pathway, associated with high CYP3A4-induced metabolism, may lead to the accumulation of 7-amino-clonazepam. This metabolite has a partial agonist effect on GABA_A_ receptor and may modify clonazepam effects. Furthermore, it is associated to withdrawal symptoms. In patients with high CYP3A4 activity and low NAT2 acetylation a longer dose reduction period may be needed [299,300]
DZP	CYP2C19	CYP2C19*1 allele is wild type (normal activity) [301]	Not required
		CYP2C19*17 allele is related to increased activity. Heterozygote individuals (*1/*17) are considered rapid metabolizers, whereas homozygote (17*/17*) are ultrarapid metabolizers [301]	Dose increase based on clinical effectiveness and holding in account other drugs taken
		CYP2C19*2 and *3 are associated with the absence of activity. Individuals with one nonfunctional allele are intermediate metabolizers. Instead of, people with 2 nonfunctional alleles are poor metabolizers [301]	Dose reduction based on clinical effectiveness and side effects. Consider other drugs taken
	CYP3A4	Several polymorphisms have been identified (e.g., CYP3A4 1*B), but their frequency or their effect on benzodiazepines is not clear. In general isoform activity seem to be preserved [302,303]. Punctiform mutations may also affect CYP3A4 function and ethnicity is another important factor [304]. More than 856 CYP3A4 variants are documented [304]	
		CYP3A4*1 is related to normal function [305]	-
		CYP3A4*20 is related to loss of function. CYP3A4*22 is associated with reduced activity [305]	Unknown impact on benzodiazepines, but possible dose reduction
		CYP3A4 1*B has an uncertain effect on enzyme’s activity. Some studies described a decreased action, other an increased action (tacrolimus, ciclosporin, simvastatin) [305]	Unknown impact of benzodiazepines
		CYP3A4 1*G seem to be associated with higher substrate clearance, but further studies are needed and some controversial results are described [305]	Unknown impact of benzodiazepines, but possible dose increase
**Antihistamines**			
Class related Elements	H1 receptor	Polymorphisms in this gene (rs901865 allele) were associated to increased side effects for desloratadine, and may have similar effects on other antihistamines [306] The experience of Chu showed that H1 polymorphisms (CC genotype) were associated to a better clinical response in allergic rhinitis, whereas other polymorphisms (CT + TT) generated a worse effect [307]	Further studies needed in vestibular diseases
	H4 receptor	The rs77041280 mutant genotype (TA + TT) and the rs77485247 mutant genotype (TA + AA) may lower drug effects and enhance adverse events after using antihistamines for allergic rhinitis [308]	Further studies needed in vestibular diseases
	C5AR1	Polymorphisms of this protein (-1330 T/G) seem to be a predictor of H1 antihistamines efficacy in urticaria [309]	Further studies needed
DPH	CYP2D6	CYP2D6*1, *2, *33, *35 alleles generate a normal function CYP2D6. On the basis of alleles homo-heterozygosis patients may be classified in ultrarapid metabolizers, extensive (or normal) metabolizers, intermediate metabolizers, and poor metabolizers [310] Zhang et al. described some example of genotype related to a specific phenotype [311]	No dose adjustment in homozygosis. Evaluate in case of heterozygosis
		CYP2D6*3, *4, *5, *6, *7,*8, *11, *12, *13, *14,*15, *16, *18, *19, *20, *21, *38, *40, *42 are examples of nonfunctional alleles [310].	Dose reduction may be needed, depending on homo-heterozygosis
		*9, *10, *17, *36, *41 alleles have decreased function [310].	Dose reduction may be needed, depending on homo-heterozygosis
		CYP2D6 *1×N, *2×N, *35×N are associated with increased activity [310]	Dose increase may be needed, depending on homo-heterozygosis. In ultrarapid metabolizers DPH generated paradoxical excitation [312]
MCZ	CYP2D6	No dedicated literature on meclizine (see DPH section for CYP2D6 polymorphisms possible influence)	
**Anticholinergics**			
Class related Elements	Muscarinic receptors	There are documented polymorphisms of M_1_ and M_2_ in literature, whereas M_3_ variants are less known. No clear link between phenotype and genotype has been described. Implications on drug responsiveness is not available [313]	Further studies needed
**Other drugs**			
MCP	CYP2D6	CYPD6 *1 and *2 represent wild type variants. Genotype CYP2D6*wild type/wild type is associated with normal enzyme activity (for further information on single CYP2D6 alleles, see DPH section) [98]	No dose adjustment
		CYP2D6 *wild type/*10, *5/*10, and *10/*10 were associated with reduced activity and higher metoclopramide levels [98]	Dose reduction
	CYP3A4, CYP2C19	No dedicated literature on MCP (see previous sections for CYP3A4 and CYP2C19 main polymorphisms)	
	CYP1A2	It has a minor role on MCP metabolism. Koonrungsesomboonet al. performed a systematic review evaluating polymorphisms involved in CYP1A2 activity [314]	
	KCNH2	This gene determines the production of a subunit of a potassium channel, whom mechanism is voltage dependent. KCNH2 seems to be a predictive factor for MCP response or side effects development. The drug may exert its action on potassium channels [315]	Adequate dosage on the basis of different polymorphisms. Further studies needed
	ADRAID	ADRAID codifies α1_D_ adrenergic receptor, that may be a target of MCP or matter of interaction with dopaminergic system. It seems to be related to MCP clinical efficacy [315].	Adequate dosage on the basis of different polymorphisms. Further studies needed
	HTR4	HTR4 is responsible of 5HT4 receptor production. MCP is a partial agonist of this target. HTR4 polymorphisms are associated with MCP side effects [315]	Adequate dosage on the basis of different polymorphisms. Further studies needed
OND	CYP2D6	Genotypes *1/*1, *1/*2, *1/*4, *1/*5, *1/*9, *1/*41,*2/*2,*41/*41 are associated with normal activity (further information on single alleles in previous sections) [316] In general, genetic deficits may be compensated by other CYP450 isoforms [76]	No dose adjustment
		Genotypes *3/*4,*4/*4, *5/*5, *5/*6 are related to poor metabolism [316]	Few studies available, possible dose reduction
		Genotypes *4/*10,*4/*41, *5/*9 are associated with intermediate metabolism [316]	Few studies available, possible dose reduction
		Genotypes 1/*1xN, *1/*2×N, *2/*2×N are related to ultrarapid metabolism [316]	Choose a drug not metabolized by CYP2D6 or dose increase [316]
	CYP3A4	No dedicated literature available (see previous sections for CYP3A4 polymorphisms)	
	CYP1A2	CYP1A2*1A is the wild type allele in vitro and in vivo [317]	No dose adjustment
		CYP1A2*1F has been associated with increased inducibility (in vitro) [317,318]	Possible dose increase, but further studies needed
		CYP1A2*1K, *3, * 4, *6 and*7 (in vitro) are associated with abolished (*6) or decreased activity [317,319] CYP1A2*8, *11, *15, *16 have the same effect in vivo [317]	Possible dose reduction but further studies are needed
	OCT1	Tzvetkov et al. showed that OCT1 polymorphisms resulting in a loss of function may moderately increase ondansetron concentration, efficacy, and side effects [320]	Evaluate OCT1 polymorphisms in patients with CYP2D6 ultrarapid metabolism may be useful to predict clinical response [320]
	P-gp	CG haplotype (C3435TandG2677T) of P-gp was associated to an increased risk of nausea and vomiting in cancer. 3435C > T polymorphism was related to efficacy in the early phase of chemotherapy. TT genotype was associated to a higher effectiveness. CTG(C3435T, C1236T, and G2677T) patients suffered for nausea and vomiting with a major frequency [321]	Evaluate dose increase or shift to other drugs. Further studies needed
	5HT3_B_ receptor	Polymorphisms of this protein are associated with different clinical effect. Deletions-100-102delAAG of the promotor region are characterized by worse clinical effect [322]	Evaluate dose increase or shift to other drugs. Further studies needed

CLZ, clonazepam; CYP450, cytochrome 450; C5AR, component 5a receptor; DPH, diphenhydramine; DZP, diazepam; GABA, gamma-aminobutyric acid; MCP, metoclopramide; MEC, meclizine; NAT, N-acetyltransferase; OCT, organic cation transporter; OND, ondansetron; P-gp, p-glycoprotein.
